# Monitoring alpha-cypermethrin susceptibility of *Phlebotomus argentipes*, the vector of visceral leishmaniasis in India, using the CDC bottle bioassay

**DOI:** 10.1186/s13071-024-06579-w

**Published:** 2024-12-05

**Authors:** Rahul Chaubey, Ashish Shukla, Anurag Kumar Kushwaha, Shakti Kumar Singh, Om Prakash Singh, Rajiv Kumar, Phillip Lawyer, Edgar Rowton, Christine A. Petersen, Scott A. Bernhardt, Shyam Sundar

**Affiliations:** 1 Kala-Azar Medical Research Center (KAMRC), Muzaffarpur, Bihar India; 2 Department of Medicine, Institute of Medical Sciences, Banaras Hindu University, Varanasi, India; 3 Department of Biochemistry, Institute of Science, Banaras Hindu University, Varanasi, India; 4 National Museum of Natural History, Ministry of Environment Forest and Climate Change, New Delhi, India; 5 Centre of Experimental Medicine & Surgery, Institute of Medical Sciences, Banaras Hindu University, Varanasi, India; 6 Arthropod Collections, Monte L. Bean Life Science Museum, Brigham Young University, Provo, UT USA; 7 Division of Entomology, Walter Reed Army Institute of Research, Silver Spring, MD USA; 8 Department of Epidemiology, College of Public Health, University of Iowa, Iowa City, Iowa USA; 9 Center for Emerging Infectious Diseases, University of Iowa, Coralville, IA USA; 10 Department of Biology, Utah State University, Logan, UT USA

**Keywords:** *Phlebotomus argentipes*, Diagnostic dose and exposure time, Insecticide susceptibility, CDC bottle bioassay

## Abstract

**Background:**

Visceral leishmaniasis (VL), known as Kala-azar on the Indian subcontinent, is a parasitic disease caused by the flagellated protozoa *Leishmania donovani* and can be fatal if left untreated. The sand fly *Phlebotomus argentipes* is the only proven vector of VL in the Southeast Asia region, and VL control in this region has relied on the use of synthetic insecticides for indoor residual spraying (IRS). The use of DDT in VL control programmes has led to the development of resistance to this insecticide in sand flies, resulting in DDT being replaced with the insecticide alpha-cypermethrin. However, alpha-cypermethrin has a similar mode of action as DDT and, therefore, the risk of resistance development in sand flies increases under the pressure of regular exposure to this insecticide. In the present study we assessed the susceptibility status of wild-caught sand flies and F1 progeny using the CDC bottle bioassay.

**Methods:**

Sand flies were collected from 10 villages in Muzaffarpur District, Bihar, India. Eight of these villages are receiving continuous IRS with alpha-cypermethrin, one village had discontinued IRS with alpha-cypermethrin and one village had never received IRS with alpha-cypermethrin. The collected sand flies were exposed to a pre-determined diagnostic dose for a specific time duration (3 µg/ml for 40 min), and knockdown and mortality at 24 h post-exposure were recorded.

**Results:**

Knockdown ranged from 91.19% to 99.47% for wild-caught sand flies and from 91.70% to 98.89% for their F1 progeny. At 24 h post-exposure, mortality ranged from 89.34% to 98.93% for wild-caught sand flies and from 90.16% to 98.33% for F1 progeny.

**Conclusions:**

The results of this study showed that *P. argentipes* is potentially developing resistance, signalling the need for continuous monitoring and vigilance to sustain the validation of elimination once achieved.

**Graphical Abstract:**

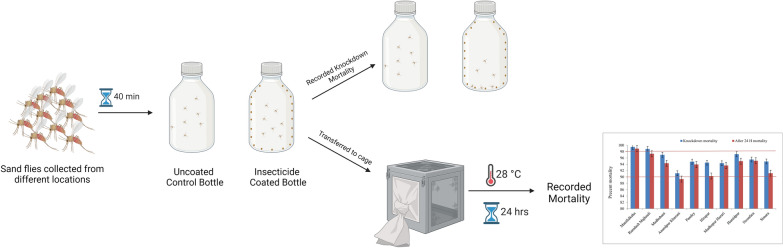

## Background

Visceral leishmaniasis (VL), known as Kala-azar on the Indian subcontinent, is a parasitic disease caused by the flagellated protozoa *Leishmania donovani* and transmitted by bites of infected female sand flies *Phlebotomus argentipes* (Diptera: Psychodidae). *Phlebotomus argentipes* is the only proven vector of VL in the Southeast Asia region. The goal of total VL elimination is very close to being achieved in India. However, to maintain low case levels post total elimination, it is critical to reduce vector populations to prevent potential transmission.

Sand fly control in the Southeast Asia region has relied on the use of synthetic insecticides for indoor residual spraying (IRS). The sheltered resting behaviour of *P. argentipes* makes them suitable targets for control by IRS with insecticides [1]. IRS undertaken within the framework of the Indian National Malaria Control Program using dichlorodiphenyltrichloroethane (DDT) had immense collateral benefits by controlling sand fly populations and significantly reducing VL cases [2]. This unplanned control of VL led to the adoption of IRS by the Indian VL Elimination Program as the main focus for *P. argentipes* control. In 2005, the governments of India, Bangladesh and Nepal signed a memorandum of understanding for the elimination of VL by 2015 [3]. The elimination effort consisted of using a combination of vector control, rapid diagnosis and treatment of human cases, with the goal of reaching the consolidation phase by 2015, which was subsequently revised to 2017 and then to 2020 [4]. A new global road map for the elimination of neglected tropical diseases, includes elimination of VL by 2030 [5].

Sand flies were considered to be susceptible to insecticides until the first report of resistance in *Phlebotomus papatasi* in 1978 [6], followed by documented resistance in *P. argentipes* in 1993 [7], a year after the third cycle of DDT was used to prevent VL in India. Thereafter, periodic studies on the susceptibility of phlebotomine sand flies to DDT and other groups of insecticides were conducted, and susceptible, tolerant and resistant populations from endemic and non-endemic regions of the Indian subcontinent were reported [2, 8–15]. Coleman et al. [2] reported a decreasing trend in *P. argentipes* susceptibility to DDT in Bihar State over the past 22 years and concluded that DDT-based IRS was suboptimal for achieving the goal of VL elimination. Given the paramount importance of IRS to VL control, insecticide resistance poses a very real threat to achieving and sustaining the elimination goals. With declining DDT effectiveness for sand fly control, a synthetic pyrethroid (SP), alpha-cypermethrin (5% wettable powder [WP]), was introduced as an alternative in the second phase of IRS [16].

Two-point mutations in the knockdown resistance gene (*kdr*) gene at codon 1014 (L1014F and L1014S) in DDT-resistant *P. argentipes* have been documented that exert a strong effect on the development of DDT resistance [17]. However, very little information is available on the susceptibility of *P. argentipes* to alpha-cypermethrin in India. Previous reports of DDT resistance have provided a clue for possible cross-resistance to alpha-cypermethrin due to the involvement of kdr mutations at a common target site, the voltage-gated sodium channel gene (*Vgsc*) in nerve cells [17]. Recently, sand flies have been found that were resistant (mortality rate < 90%) to alpha-cypermethrin and possibly resistant (mortality rate 90–97%) to the insecticides deltamethrin and lambda-cyhalothrin in IRS villages in Nepal [18], along with minimal possible resistance being detected to alpha-cypermethrin in sand flies in India, with mortality ranging from 97.6% to 100% [19].

As India enters the post-elimination phase of the VL elimination programme, it will be critical to ensure that resistance does not significantly develop against alpha-cypermethrin. This potential for resistance is due to both DDT and alph-cypermethrin having the same mode of action of targeting the VGSC protein [21]. Thus, the risk of resistance development in sand flies has the potential to increase under the pressure of regular exposure to alpha-cypermethrin. It is therefore critical to conduct surveillance and identify potential sand fly populations that are resistant to this insecticide. In this context, the aim of the present study was to monitor the susceptibility status of wild-caught *P. argentipes*, using the diagnostic dose and exposure time established by Chaubey et al. [20], from different villages of Muzaffarpur district, Bihar State, India that have been receiving continuous IRS with alpha-cypermethrin (continuous IRS villages) and compare the susceptibility status with that of wild-caught *P. argentipes* from one village that had discontinued IRS with alpha-cypermethrin (former IRS village) and from one village that has never received IRS with alpha-cypermethrin (non-IRS village), using the CDC bottle bioassay.

## Methods

### Study sites

**Fig. 1:**
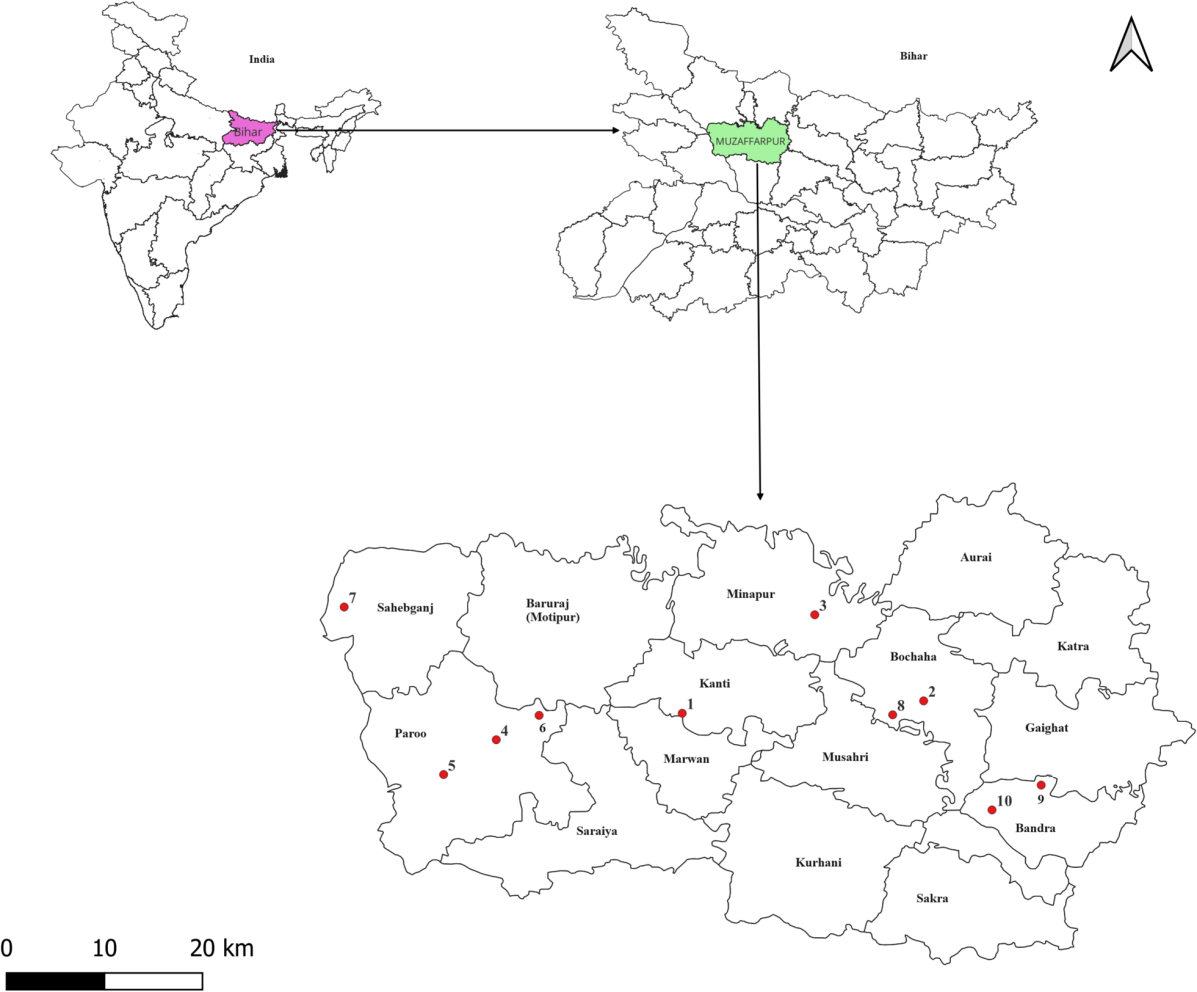
Geographical map of Muzaffarpur district showing the locations (1–10) of the villages included in the study. Study sites: 1, Manifulkaha; 2, Ramdas Majhauli; 3, Madhubani; 4, Anandpur Kharuni; 5, Pandey; 6, Hirapur; 7, Madhopur Hazari; 8, Hamidpur; 9, Noonfara; 10, Simara. The map was produced using QGIS software (version 3.30.3), with open assess shapefile (https://onlinemaps.surveyofindia.gov.in).

Ten villages were selected for inclusion in the study (Fig. 1; Table 1), of which eight have a history of receiving continuous IRS with synthetic pyrethroids (alpha-cypermethrin; denoted as continuous IRS villages) and had VL cases (at least 1 case) over the last 3 years. Of the two remaining villages included in the study, one village with no history of IRS with alpha-cypermethrin (non-IRS village) was selected as a control, and one village with a history of discontinuous IRS with alpha-cypermethrin (discontinuous IRS village/former IRS village) was selected. Selection of these villages was based on coordination with the Ministry of Health and IRS team and confirmation with the micro action plan for IRS in Muzaffarpur District.

**Table 1:** Detailed description of the 10 villages selected for inclusion in the study

Village	Latitude	Longitude	Number of VL cases reported					IRS with SP performed in:
			2017	2018	2019	2020	2021	
Manifulkaha^a^	26.15934	85.24143						
Ramdas Majhauli^b^	26.17192	85.48724		1				2017, 2018, 2019, 2020, 2021
Madhubani	26.25955	85.37637	2	5	3	2		2017, 2018, 2019, 2020, 2021, 2022
Anandpur Kharuni	26.13238	85.05207	4	23	8	6	3	2017, 2018, 2019, 2020, 2021, 2022
Pandey	26.09698	84.99848	1	16	9	2	3	2017, 2018, 2019, 2020, 2021, 2022
Hirapur	26.15715	85.09567	6	6	1	2	3	2017, 2018, 2019, 2020, 2021, 2022
Madhopur Hazari	26.26743	84.89722	18	32	13	2	1	2017, 2018, 2019, 2020, 2021, 2022
Hamidpur	26.15774	85.45569	8	2	2	3	1	2017, 2018, 2019, 2020, 2021, 2022
Noonfara	26.08619	85.60692	3	2	2	5	1	2017, 2018, 2019, 2020, 2021, 2022
Simara	26.06095	85.55684	3	3	2	1	1	2017, 2018, 2019, 2020, 2021, 2022

Villages were selected for inclusion in the study based on continuous case report and IRS with the synthetic pyrethroid (SP) alpha-cypermethrin for at least 3 years

*IRS* Indoor residual spraying, *SP* alpha-cypermethrin,*VL* visceral leishmaniasis

^a^Non-IRS village (village with no history of receiving IRS with alpha-cypermethrinof)

^b^Discontinuous IRS village/former IRS village (village that had discontinued IRS with alpha-cypermethrin)

### Sand fly collection

Monthly sand fly collections using CDC light traps were conducted in the selected villages following the methods of Tiwary et al. [21]. Briefly, light traps were installed in the evening at 6:00 p.m. and collected the next morning at 6:00 a.m. The traps were then transported to the insectary at the Kala-Azar Medical Research Center (KAMRC) where the live sand flies were sorted with a mouth aspirator and transferred to small acrylic holding cages. All blood-fed and gravid females were placed individually in isoline vials for egg-laying and subsequent rearing following the methods of Tiwary et al. [21]. The holding cages with the remaining live flies were kept in the environmental chamber and provided with a cotton ball soaked in a 30% sugar solution and apple slices (placed on the screen-mesh tops of the cages) as food/energy sources. The environmental chamber was maintained at 27 °C and 80% relative humidity for 24 h to acclimatize the flies and reduce stress. The next morning, surviving unfed female *P. argentipes* sand flies were exposed to insecticide for assessing the susceptibility status.

### Preparation of exposure bottle

Bottles for the exposure experiment were prepared following the methods of Chaubey et al. [20] and Denlinger et al. [22]. Briefly, the day prior to the experiment, 500-ml glass bottles were prepared by coating them on the inside with the designated insecticide (diagnostic dose for alpha-cypermethrin is 3 µg/ml) by swirling the acetone-insecticide solution (2.0 ml) on the bottom, sides and lid of the bottle. Each bottle was then placed on a mechanical roller for 30 min to dry. During this time the lids were slowly loosened to allow the acetone to evaporate. After 30 min of drying, the lids were removed, and the bottles rolled around until all of the acetone had completely evaporated. The bottles were then left open to dry overnight. For each test replicate, one bottle serving as the control was coated with 2.0 ml of acetone. All bottles were reused throughout the experiment after proper cleaning following the procedure described in Denlinger et al. and WHO [22, 23].

### Bioassay and susceptibility analysis

The day after the bottles were prepared with insecticide, 30–40 wild-caught sand flies (unfed females) were aspirated from the cage and gently blown into each bottle. Approximately the same number of flies was utilized for each insecticide-coated bottle, including the control bottle. There were a minimum of five to six replications for each village. After 40 min of insecticide exposure, the number of knockdown flies was recorded. All flies were captured with a mechanical aspirator, released into a 1-pint cardboard container with a fine mesh screen covering the top and maintained in a separate incubator under the same humidity and temperature conditions and with the same food source (cotton ball soaked in 30% sugar solution) as the untreated colony. Mortality was recorded 24 h after insecticide exposure. All sand flies were dissected and examined for species confirmation. The same followed for the F1 progeny flies. The knockdown and 24 h post-exposure mortality were recorded. The mortality correction was not used for the test replicate if the mortality in the control bottle was < 5%. If mortality in the control bottle was ≥ 5% and ≤ 20%, mortality in the test bottles of that replicate was corrected using Abbott’s formula. If the control mortality exceeded 20%, the entire test group was discarded [24, 25, 26].

## Results

**Fig. 2:**
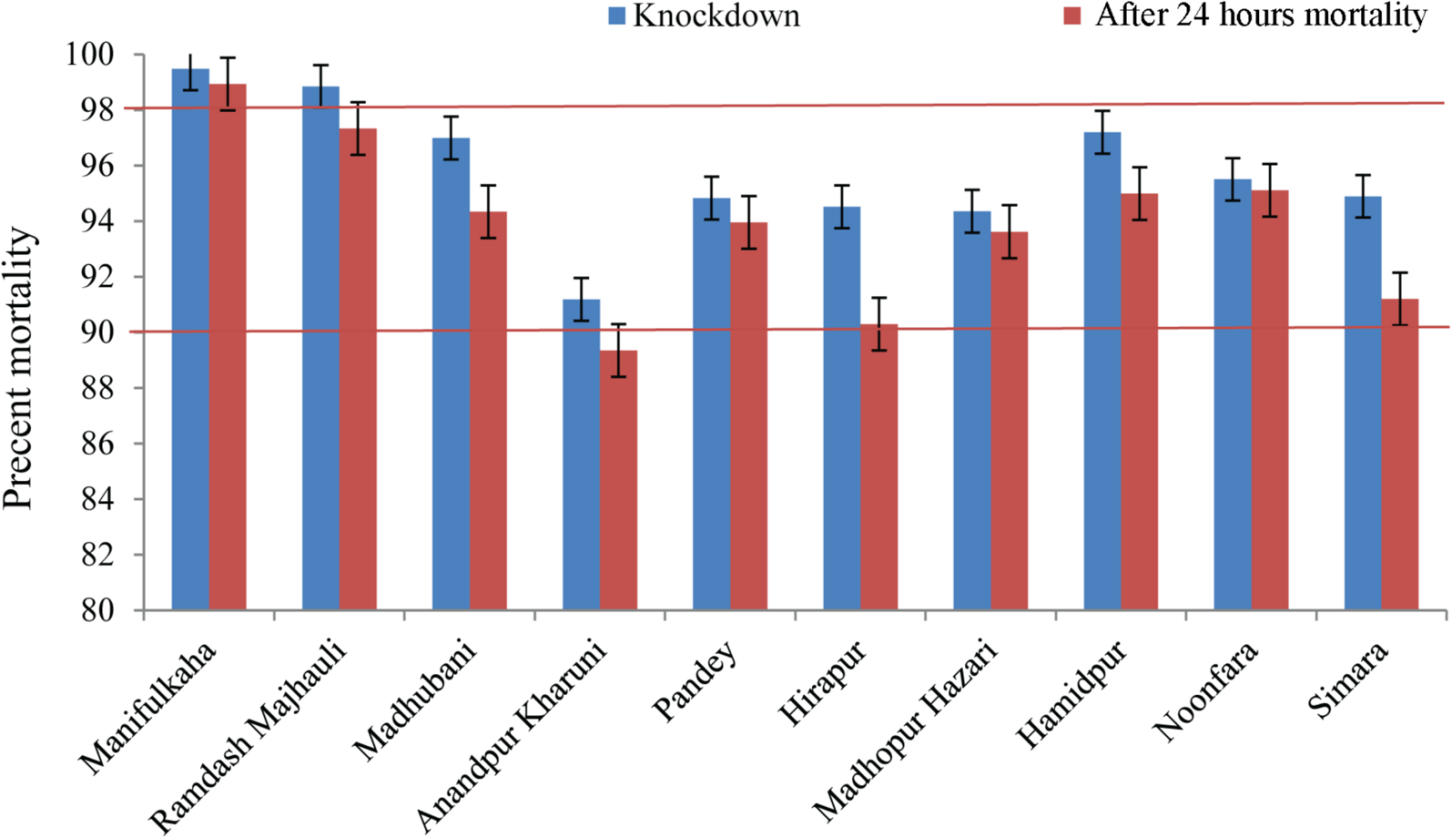
Mean percent mortality of field-caught *Phlebotomus argentipes* sand flies. Error bars represent the standard error of the mean. The two red horizontal lines intercept the graph (at mortality levels of 90% and 98%) represent the percent mortality window for possible resistance [25]

**Fig. 3:**
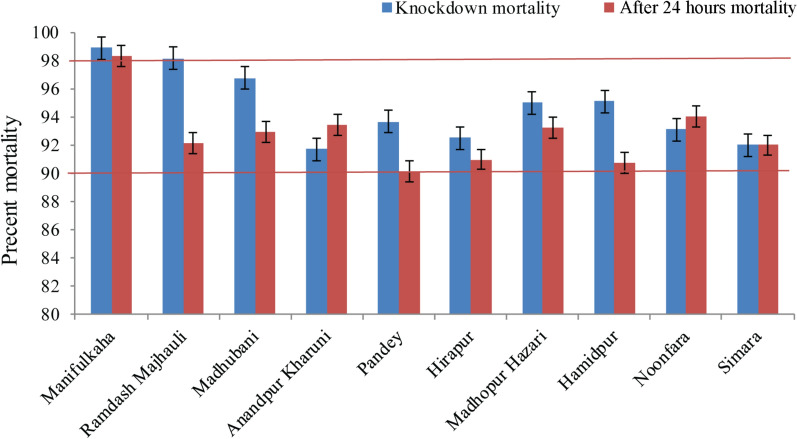
Mean percent mortality of field-caught *Phlebotomus argentipes* F1 progeny. Error bars represent the standard error of the mean. The two red horizontal lines intercept the graph (at mortality levels of 90% and 98%) represent the percent mortality window for possible resistance [25]

The sand flies *P. argentipes* from the continuous IRS villages were found to be tolerant/possibly resistant, with mean (± standard error [SE]) mortality in the wild-caught flies ranging from 91.19 ± 1.71% to 97.20 ± 0.61% at knockdown and from 90.30 ± 0.71% to 95.11 ± 3.18% at 24 h post-exposure (referred to as 24 h mortality) (Table 2; Fig. 2). The mean mortality of F1 progeny was recorded as ranging from 91.70 ± 0.69% to 95.11 ± 2.68% at knockdown and from 90.16 ± 1.82% to 94.00 ± 2.94% at 24 h post-exposure (Table 3, Fig. 3). The 24 h mortality of wild-caught sand flies from Anandpur Kharuni (89.34 ± 2.52%) was recorded as indicating resistance (Table 2; Fig. 2).

**Table 2:** Percent mortality of field-caught *Phlebotomus argentipes* at the diagnostic dose and pre-determined exposure time for alpha-cypermethrin from different villages using the CDC bottle bioassay

Village	No. of sand flies tested	Mortality at knockdown (%)^a^	Mortality at 24 h post-exposure (%)^a^
Manifulkaha	134	99.47 ± 0.52	98.93 ± 0.65
Ramdas Majhauli	145	98.85 ± 0.73	97.33 ± 1.33
Madhubani	256	96.99 ± 0.45	94.35 ± 0.76
Anandpur Kharuni	95	91.19 ± 1.71	89.34 ± 2.52
Pandey	219	94.83 ± 1.54	93.96 ± 2.51
Hirapur	160	94.52 ± 2.18	90.30 ± 0.71
Madhopur Hazari	206	94.36 ± 0.97	93.62 ± 1.85
Hamidpur	185	97.20 ± 0.61	94.99 ± 1.45
Noonfara	141	95.50 ± 1.99	95.11 ± 3.18
Simara	95	96.99 ± 2.11	94.35 ± 3.68

The diagnostic dose and exposure time were 3 µg/ml and 40 min, respectively

^a^Values are reported as the mean ± standard error

**Table 3:** Percent mortality of F1 progeny of filed-caught *P. argentipes* at the diagnostic dose and pre-determined exposure time for alpha-cypermethrin from different villages using the CDC bottle bioassay

Village	No. of sand flies tested	Mortality at knockdown (%)^a^	Mortality at 24 h post-exposure^a^
Manifulkaha	137	98.89 ± 1.11	98.33 ± 1.11
Ramdas Majhauli	145	98.16 ± 1.37	92.13 ± 1.61
Madhubani	129	94.98 ± 1.54	93.21 ± 2.17
Anandpur Kharuni	158	91.70 ± 0.69	93.42 ± 1.31
Pandey	178	93.68 ± 1.34	90.16 ± 1.82
Hirapur	167	92.50 ± 2.13	90.97 ± 0.94
Madhopur Hazari	111	94.98 ± 0.81	93.21 ± 2.12
Hamidpur	135	95.11 ± 2.68	90.77 ± 2.68
Noonfara	115	93.12 ± 1.78	94.00 ± 2.94
Simara	51	92.00 ± 3.92	92.00 ± 3.92

The diagnostic dose and exposure time were 3 µg/ml and 40 min, respectively

^a^Values are reported as the mean ± standard error

The wild-caught sand flies from the former IRS village/discontinuous IRS village (Ramdas Majhauli) were found to be susceptible (mean [± SE] mortality at knockdown 98.85 ± 0.73%; mean mortality at 24 h post-exposure 97.33 ± 1.33%) (Table 2; Fig. 2). The mean mortality of F1 progeny at knockdown (98.16 ± 1.37%) also revealed susceptibility, but mortality at 24 h post-exposure (92.13 ± 1.61%) was recorded as possibly resistant (Table 3; Fig. 3).

Sand flies from the control/non-IRS village (Manifulkaha) were found to be highly susceptible to the insecticide. The mean (± SE) mortality of wild-caught sand flies at knockdown and at 24 h post-exposure was 99.47 ± 0.52% and 98.93 ± 0.65%, respectively, and that of the F1 progeny was 98.89 ± 1.11% and 98.33 ± 1.11%, respectively) (Tables 2, 3), respectively.

## Discussion

The results of this study showed that *P. argentipes* is starting to develop possible resistance to the synthetic pyrethroid (SP) alpha-cypermethrin in those villages that have received continuous IRS with this SP. In comparison, the *P. argentipes* caught in the non-IRS/control village were highly susceptible. Monitoring the susceptibility status of wild *P. argentipes* populations is essential in order to monitor the effectiveness of the insecticides being used, as this information can help in insecticide resistance management. Sand flies in the endemic areas of Bihar State are periodically reported as highly resistance to DDT, a result of the historical selection pressure exerted by IRS rounds with this insecticide [1].

*Phlebotomus argentipes* was found to be highly susceptible to pyrethroids, and field trials carried out in India, Bangladesh and Nepal have shown high entomological efficacy of IRS with alpha-cypermethrin or deltamethrin [19, 26–29]. Recently, Roy et al. [18] reported from Nepal that *P. argentipes* had developed resistance against pyrethroids. Our field susceptibility study showed that *P. argentipes* collected from the non-IRS village are highly susceptible, but the flies collected from the discontinuous IRS/former IRS and continuous IRS villages (mortality of between 90% and 97%, with the exception of sand flies from Anandpur Kharuni at 24 h post-exposure: 89.34% mortality) are developing possible resistance to alpha-cypermethrin [25]. One possible reason for the development of this resistance is the pressure exerted by routine IRS programmes and case-based focal spraying, the standard procedure for managing Kala-azar outbreaks in endemic districts/blocks/villages (standard operating procedure for outbreak investigation and management [30]. The results of the present study provide evidence of an early sign of the development of selection pressure against alpha-cypermethrin. Unfortunately, historical susceptibility data from this region using the CDC bottle bioassay are not available for comparison; all previous studies monitored susceptibility of *P. argentipes* using the WHO insecticide impregnated papers. The diagnostic doses of insecticides in WHO impregnated test papers are the discriminating concentrations of the insecticides recommended for malaria vectors (*Anopheles* mosquitoes) and the operational applicability of these concentrations for sand flies is unclear because sand flies are apt to fly less than mosquitoes in bioassays and spend more time in contact with the substrate [23].

IRS with synthetic pyrethroids has been used to control sand flies in VL endemic districts of Nepal since 1992 by alternating the use of the SPs alpha-cypermethrin and lambda-cyhalothrin [31], and in Bangladesh since 2012 with deltamethrin [32]. Phenotypic resistance has been detected in the wild populations of *P. argentipes* in areas where synthetic pyrethroids have long been applied [18, 33, 34]. The presence of a non-synonymous mutation (L1014F) in the wild population of *P. argentipes* in India, which was found to be associated with resistance to DDT, signals the emergence of pyrethroid resistance at the molecular level because DDT and pyrethroids (alpha-cypermethrin) both target the same gene in the insect nervous system [17, 34]. Therefore, systematic susceptibility evaluation and resistance monitoring for alpha cypermethrin in sand flies are very much needed in the VL elimination/post elimination phase.

A potential limitation of this study is that we used the CDC bottle bioassay to measure susceptibility, but all comparisons used results from previous studies that used the WHO test kit bioassay. The results of the two bioassay methods may not be directly comparable as the CDC bottle bioassays measure knockdown at the end of a diagnostic time, whereas the WHO test kit bioassays measure mortality at 24 or 72 h post-exposure (the latter time for slow-acting compounds) [35]. Another potential limitation is the number of IRS villages in this study in comparison to one non-IRS village and one discontinuous IRS/former IRS village. We cannot assume that the susceptibility status of the sand fly vectors observed in the selected village from one district is representative of that of the other villages and districts of Bihar State. As India enters the post-elimination phase of VL, it will be critical to ensure that resistance does not develop significantly. Operational monitoring of resistance in sand fly populations is needed from different districts, blocks and geographic areas. The data presented in this study are very preliminary and need to be validated by comparison with the discriminating concentrations published by WHO [35] in order to be more specific regarding the susceptibility status of *P. argentipes* in these regions before vector control protocols are modified to keep sand fly populations low and maintain VL elimination.

## Conclusions

The vector of VL, the sand fly *P. argentipes*, is possibly starting to show early signs of resistance to alpha-cypermethrin. Regular monitoring of insecticide resistance in wild populations of *P. argentipes* is warranted to maintain the epidemiological impact of vector control interventions. Alternating insecticides with different modes of actions and/or new insecticide evaluation and registration are needed and recommended for insecticide-resistance management to maintain and sustain the VL elimination in India.

## Data Availability

No datasets were generated or analysed during the current study.

## References

[1] Dhiman RC, Yadav RS. Insecticide resistance in phlebotomine sandflies in Southeast Asia with emphasis on the Indian subcontinent. Infect Dis Poverty. 2016;5:1–10. 27817749 10.1186/s40249-016-0200-3 PMC5098277

[2] Coleman M, Foster GM, Deb R, Singh RP, Ismail HM, Shivam P, et al. DDT-based indoor residual spraying suboptimal for visceral leishmaniasis elimination in India. Proc Natl Acad Sci USA. 2015;112:8573–8. 26124110 10.1073/pnas.1507782112 PMC4507214

[3] Kumar V, Kesari S, Kumar AJ, Dinesh DS, Ranjan A, Prasad M, et al. Vector density and the control of Kala-azar in Bihar. India Mem Inst Oswaldo Cruz. 2009;104:1019–22. 20027471 10.1590/s0074-02762009000700014

[4] WHO. Independent assessment of Kala-Azar elimination programme India. Geneva: World Health Organization; 2019.

[5] WHO. Ending the neglect to attain the sustainable development goals: a road map for neglected tropical diseases 2021–2030. Geneva: WHO; 2020.

[6] Kaul SM, Wattal BL, Bhatnagar VN, Mathur KK. Preliminary observations on the susceptibility status of Phlebotomus argentipes and P. papatasi to DDT in two districts of North Bihar (India). J Commun Dis. 1978;10:208–11.

[7] Kaul SM, Das RK, Shiv Raj SR, Saxena NBL, Narasimham MVVL. Entomological monitoring of Kala-azar control in Bihar State, India: observations in Vaishali and Patna Districts. J Commun Dis. 1993;25:101–6.

[8] Joshi GC, Kaul SM, Wattal BL. Susceptibility of sandflies to organochlorine insecticides in Bihar (India)—further reports. J Commun Dis. 1979;11:209–13.

[9] Dhanda V, Shetty PS, Dhiman RC. Studies on phlebotomine sandflies as vectors of Kala-azar in Bihar. In: Proceedings of Indo-UK Workshop on Leishmaniasis. Indian Council of Medical Research: New Delhi; 1983. p. 128–37.

[10] Das Gupta RK, Saxena NB, Joshi RD, Rao JS. DDT resistance in* P. papatasi* in Bihar. J Commun Dis. 1995;27:124. 7499773

[11] Singh KV, Bansal SK. Insecticide susceptibility of* Phlebotomus papatasi* to organochlorine, organophosphate & carbamate compounds in some arid areas of western Rajasthan. Indian J Med Res. 1996;103:91–3. 8714145

[12] Amalraj DD, Sivagnaname N, Srinivasan R. Susceptibility of* Phlebotomus argentipes* and* P. papatasi *(Diptera : Psychodidae) to insecticides. J Commun Dis. 1999;31:177–80. 10916614

[13] Dhiman RC, Mittal PK. A note on susceptibility status of* Phlebotomus papatasi* (Scopoli) populations to insecticides. J Commun Dis. 2000;32:65–6. 11129568

[14] Dhiman RC, Raghavendra K, Kumar V, Kesari S, Kishore K. Susceptibility status of* Phlebotomus argentipes* to insecticides in districts Vaishaii and Patna (Bihar). J Commun Dis. 2003;35:49–51. 15239308

[15] Dinesh DS, Das ML, Picado A, Roy L, Rijal S, Singh SP, et al. Insecticide susceptibility of* Phlebotomus argentipes* in visceral leishmaniasis endemic districts in India and Nepal. PLoS Negl Trop Dis. 2010;4:e859. 21049013 10.1371/journal.pntd.0000859 PMC2964302

[16] WHO. Kala-azar elimination programme. Report of WHO Consultation of Partners. Geneva 10–11 February 2015. Geneva:WHO; 2015.

[17] Gomes B, Purkait B, Deb RM, Rama A, Singh RP, Foster GM, et al. Knockdown resistance mutations predict DDT resistance and pyrethroid tolerance in the visceral leishmaniasis vector* Phlebotomus argentipes*. PLoS Negl Trop Dis. 2017;11:1–14. 10.1371/journal.pntd.0005504 PMC5407848 28414744

[18] Roy L, Uranw S, Cloots K, Smekens T, Kiran U, Pyakurel UR, et al. Susceptibility status of the wild-caught* Phlebotomus argentipes* (Diptera: Psychodidae: Phlebotominae), the sand fly vector of visceral leishmaniasis, to different insecticides in Nepal. PLoS Negl Trop Dis. 2022;16:1–18. 10.1371/journal.pntd.0010304 PMC9321455 35834563

[19] Deb R, Singh RP, Mishra PK, Hitchins L, Reid E, Barwa AM, et al. Impact of IRS: four-years of entomological surveillance of the indian visceral leishmaniases elimination programme. PLoS Negl Trop Dis. 2021;15:1–19. 10.1371/journal.pntd.0009101 PMC8376195 34370731

[20] Chaubey R, Shukla A, Kushwaha AK, Tiwary P, Singh SK, Hennings S, et al. Assessing insecticide susceptibility, diagnostic dose and time for the sand fly* Phlebotomus argentipes*, the vector of visceral leishmaniasis in India, using the CDC bottle bioassay. PLoS Negl Trop Dis. 2023;17:e0011276. 37163529 10.1371/journal.pntd.0011276 PMC10202287

[21] Tiwary P, Singh SK, Kushwaha AK, Rowton E, Sacks D, Singh OP, et al. Establishing, expanding, and certifying a closed colony of* Phlebotomus argentipes* (Diptera: Psychodidae) for xenodiagnostic studies at the Kala Azar Medical Research Center, Muzaffarpur, Bihar. India J Med Entomol. 2017;54:1129–39. 28525618 10.1093/jme/tjx099 PMC5850120

[22] Denlinger DS, Lozano-Fuentes S, Lawyer PG, Black WC, Bernhardt SA. Assessing insecticide susceptibility of laboratory* Lutzomyia longipalpis* and* Phlebotomus papatasi* sand flies (Diptera: Psychodidae: Phlebotominae). J Med Entomol. 2015;52:1003–12. 26336231 10.1093/jme/tjv091 PMC4574604

[23] WHO. Operational manual on leishmaniasis vector control, surveillance, monitoring and evaluation. Geneva: WHO; 2022.

[24] Denlinger DS, Creswell JA, Anderson JL, Reese CK, Bernhardt SA. Diagnostic doses and times for *Phlebotomus papatasi* and *Lutzomyia longipalpis* sand flies (Diptera: Psychodidae: Phlebotominae) using the CDC bottle bioassay to assess insecticide resistance. Parasit Vectors. 2016;9:1. 27083417 10.1186/s13071-016-1496-3 PMC4833940

[25] WHO. Test procedures for insecticide resistance monitoring in malaria vector mosquitoes. Second edition. Global Malaria Program. Geneva: WHO; 2016.

[26] Joshi AB, Das ML, Akhter S, Chowdhury R, Mondal D, Kumar V, et al. Chemical and environmental vector control as a contribution to the elimination of visceral leishmaniasis on the Indian subcontinent: Cluster randomized controlled trials in Bangladesh, India and Nepal BMC Med. 2009;7:54. 19804620 10.1186/1741-7015-7-54 PMC2763005

[27] Chowdhury R, Dotson E, Blackstock AJ, McClintock S, Maheswary NP, Faria S, et al. Comparison of insecticide-treated nets and indoor residual spraying to control the vector of visceral leishmaniasis in Mymensingh District, Bangladesh. Am J Trop Med Hyg. 2011;84:662–7. 21540372 10.4269/ajtmh.2011.10-0682 PMC3083730

[28] Chowdhury R, Das ML, Chowdhury V, Roy L, Faria S, Priyanka J, et al. Susceptibility of field-collected *Phlebotomus argentipes* (Diptera: Psychodidae) sand flies from Bangladesh and Nepal to different insecticides. Parasit Vectors. 2018;11:1. 29866195 10.1186/s13071-018-2913-6 PMC5987452

[29] Dinesh DS, Hassan F, Kumar V, Kesari S, Topno RK, Yadav RS. Insecticide susceptibility of* Phlebotomus argentipes* sandflies, vectors of visceral leishmaniasis in India. Trop Med Int Health. 2021;26:823–8. 33733549 10.1111/tmi.13576

[30] Standard operating procedure for outbreak investigation and management. Directorate of National Vector Borne Disease Control Program, Ministry of Health and Family Welfare, Government of India; 2020.

[31] Government of Nepal, Ministry of Health and Population, Department of Health Services, Epidemiology & Disease Control Division. The internal assessment of malaria and Kala-azar elimination activities 2007, 2008 and 2009. Ministry of Health, Nepal; 2010.

[32] Chowdhury R, Mondal D, Chowdhury V, Faria S, Alvar J, Nabi SG, et al. How far are we from visceral leishmaniasis elimination in Bangladesh? An assessment of epidemiological surveillance data. PLoS Negl Trop Dis. 2014;8:1–10. 10.1371/journal.pntd.0003020 PMC4140646 25144317

[33] Karakus M, Gocmen B, özbel Y. Insecticide susceptibility status of wild-caught sand fly populations collected from two leishmaniasis endemic areas in western Turkey. J Arthropod Borne Dis. 2017;11:86–94. 29026855 PMC5629309

[34] Sardar AA, Saha P, Chatterjee M, Bera DK, Biswas P, Maji D, et al. Insecticide susceptibility status of *Phlebotomus argentipes *and polymorphisms in voltage-gated sodium channel (*vgsc*) gene in Kala-azar endemic areas of West Bengal. India Acta Trop. 2018;185:285–93. 29890155 10.1016/j.actatropica.2018.06.005

[35] WHO. Determining discriminating concentrations of insecticides for monitoring resistance in mosquitoes: report of a multi-centre laboratory study and WHO expert consultations. Geneva: WHO; 2022.

